# Personality and Other Lifelong Influences on Older‐Age Health and Wellbeing: Preliminary Findings in Two Scottish Samples

**DOI:** 10.1002/per.2068

**Published:** 2016-10-16

**Authors:** Mathew A. Harris, Caroline E. Brett, John M. Starr, Ian J. Deary, Wendy Johnson

**Affiliations:** ^1^ Centre for Cognitive Ageing and Cognitive EpidemiologyUniversity of EdinburghEdinburghUK; ^2^ Centre for Clinical Brain SciencesUniversity of EdinburghEdinburghUK; ^3^ School of Natural Sciences and PsychologyLiverpool John Moores UniversityLiverpoolUK; ^4^ Alzheimer Scotland Dementia Research CentreUniversity of EdinburghEdinburghUK

**Keywords:** personality, cognitive ability, education, health, subjective wellbeing, longitudinal study

## Abstract

Recent observations that personality traits are related to later‐life health and wellbeing have inspired considerable interest in exploring the mechanisms involved. Other factors, such as cognitive ability and education, also show longitudinal influences on health and wellbeing, but it is not yet clear how all these early‐life factors together contribute to later‐life health and wellbeing. In this preliminary study, we assessed hypothesised relations among these variables across the life course, using structural equation modelling in a sample assessed on dependability (a personality trait related to conscientiousness) in childhood, cognitive ability and social class in childhood and older age, education, and health and subjective wellbeing in older age. Our models indicated that both health and subjective wellbeing in older age were influenced by childhood IQ and social class, via education. Some older‐age personality traits mediated the effects of early‐life variables, on subjective wellbeing in particular, but childhood dependability did not show significant associations. Our results therefore did not provide evidence that childhood dependability promotes older‐age health and wellbeing, but did highlight the importance of other early‐life factors, particularly characteristics that contribute to educational attainment. Further, personality in later life may mediate the effects of early‐life factors on health and subjective wellbeing. © 2016 The Authors. *European Journal of Personality* published by John Wiley & Sons Ltd on behalf of European Association of Personality Psychology

One of the principal long‐term aims of ageing research is to improve the physical health and psychological wellbeing of older individuals. Despite this, one thing that has become clear is that many personal characteristics, behaviours and circumstances generally considered positive, when present quite early in life, are associated with older‐age health and wellbeing. But going beyond these basic associations to understand the mechanisms and natures of the individual differences in circumstances, behaviours, and characteristics that drive these associations and the degrees to which we might be able to manipulate them is less straightforward, and factors that are identified never explain all the observed variance in health and wellbeing in older age. Without greater understanding of the processes underlying these associations, the potential to identify and manipulate the sources of this remaining variance that offers some of the best opportunities to accomplish our long‐term goals is considerably limited.

### Personality as a predictor of health and wellbeing

Personality traits are examples of characteristics that have been shown to predict health outcomes. For instance, Hampson, Goldberg, Vogt, and Dubanoski (2007) studied relations between childhood measures of the Big Five personality traits and overall health 40 years later. They observed significant indirect positive associations between childhood extraversion, conscientiousness, agreeableness and intellect/imagination, and mid‐life health status, via educational attainment and/or health behaviours, as well as a direct association for conscientiousness. Conscientiousness in particular is consistently identified as a predictor of longevity (Friedman et al., 1993; Jokela et al., 2013) and health (Friedman, 2000; Bogg & Roberts, 2013; Hampson, Edmonds, Goldberg, Dubanoski, & Hillier, 2015). Weston, Hill, and Jackson (2015) focused on individual disease, studying the associations between the Big Five personality traits and subsequent risk of onset in 6904 older participants. Higher extraversion was associated with lower risk of developing high blood pressure, higher agreeableness with lower risk of developing arthritis, conscientiousness with lower risk of high blood pressure, diabetes, stroke and arthritis, and openness with lower risk of high blood pressure, heart conditions, stroke and arthritis, while higher neuroticism was associated with greater risk of developing high blood pressure, lung disease, heart conditions or arthritis. This range of disease‐specific associations shows how personality throughout earlier life may contribute to overall health in older age.

Wellbeing is intricately associated with health, but focuses on subjective experience of overall personal state, rather than on objective measures of illness, disease and incapacity. Subjective wellbeing in particular refers to an individual's own overall assessment of his or her life circumstances, often reflecting life and relationship circumstances, and mental as well as physical health (Diener, Oishi, & Lucas, 2009; Realo, Johannson, & Schmidt, 2016). Subjective wellbeing includes cognitive components, such as life satisfaction and self‐rated quality of life, as well as affective components, such as mental wellbeing (Schimmack, 2008). It may be even more closely related to earlier‐measured personality than is health, showing significant correlations with all the Big Five traits (Schmutte & Ryff, 1997; Steel, Schmidt, & Shultz, 2008), but particularly extraversion and neuroticism, or emotional stability (DeNeve & Cooper, 1998; Schimmack, Radhakrishnan, Oishi, Dzokoto, & Ahadi, 2002; Brett et al., 2012; Gale, Booth, Mõttus, Kuh, & Deary, 2013). For instance, Schimmack et al. observed correlations of .47 and −.48 between extraversion and neuroticism, respectively, and life satisfaction in their American sample of 168 university students. Vittersø (2001) argued that emotional stability in particular captured the majority of the association between personality and subjective wellbeing, with emotional stability accounting for around 34% of the variance in subjective wellbeing, compared to only 1% for extraversion. Brett et al. (2012) also found that emotional stability in particular was relatively strongly associated with self‐rated quality of life in older age. However, the association between emotional stability and subjective wellbeing may be at least partly attributable to a degree of overlap between the two constructs, both being defined in part by negative emotional experience. Still, in keeping with this, Friedman, Kern, and Reynolds (2010) found that subjective wellbeing in older age was particularly closely related to childhood neuroticism, but also significantly associated with childhood conscientiousness and agreeableness.

### Cognitive ability as a predictor of health and wellbeing

Cognitive ability has also been shown to predict later health and, to a lesser extent, wellbeing. For example, Wrulich et al. (2014) observed significant associations between intelligence at age 12 and doctor visits, sick‐leave days, self‐reported health and health‐related functionality at age 52. Others have reported longitudinal associations between earlier‐measured intelligence and physical health and longevity as well (O'Toole & Stankov, 1992; Deary, Whiteman, Starr, Whalley, & Fox, 2004; Calvin et al., 2011; Hagger‐Johnson, Mõttus, Craig, Starr, & Deary, 2012). There is also some evidence indicating that self‐reported or subjective wellbeing is related to cognitive ability (Rabbitt, Lunn, Ibrahim, Cobain, & McInnes, 2008; Voracek, 2009), although other studies suggest that older‐age wellbeing is less closely related to prior cognitive ability (Gow, Whiteman, Pattie, & Deary, 2005; Grossman, Na, Varnum, Kitayama, & Nisbett, 2013). Cognitive ability may also interact with personality traits in influencing later health and wellbeing. For example, Weiss, Gale, Batty, and Deary (2009) assessed the effects of cognitive ability and neuroticism on mortality among 4200 members of the Vietnam Experience Cohort. Low cognitive ability and high neuroticism both predicted mortality independently, but also showed a significant interaction, with individuals of lower cognitive ability and higher neuroticism being at particular risk. In a large meta‐analysis, Ackerman and Heggestad (1997) observed common variance underlying many measures of cognitive ability and personality. They suggested that correlations among measures of the two separate constructs are because of the parallel development of personality and intelligence. Chamorro‐Premuzic and Furnham (2004) argued that personality traits such as openness may motivate intellectual activities that promote cognitive development, while traits such as conscientiousness may be facilitated by positive reinforcement of success at intellectual tasks because of greater cognitive ability. The effects of the two on later outcomes such as health and wellbeing may be related to one another—just as, for instance, personality and cognitive ability contribute together to educational attainment (Di Fabio & Busoni, 2007; Chamorro‐Premuzic & Furnham, 2008).

### Education and social class as predictors of health and wellbeing

Furthermore, both health and wellbeing also vary with education (Ross & Wu, 1995; Cutler & Lleras‐Muney, 2006, 2010; Brunello, Fort, Schneeweis, & Winter‐Ebmer, 2015). Meeks and Murrell (2001), for instance, studied 1177 participants at a mean age of 67.4 years, and then three more times at 6‐month intervals. They observed correlations of .39 to .42 between educational attainment and health, and of .40 to .41 between education and life satisfaction. Furthermore, educational attainment is associated with cognitive ability and also related to personality; childhood personality is associated with later educational performance (e.g. De Raad & Schouwenburg, 1996), suggesting influence on it, but much of personality development takes place throughout childhood and adolescence (Caspi & Roberts, 2001), during ongoing full‐time education, so education may also influence later personality. Some of the aforementioned studies of the relations between personality and later health and wellbeing suggested that education is also involved. For example, Bogg and Roberts (2013) observed that childhood conscientiousness predicted educational attainment, which in turn predicted mid‐life health. Hampson et al. (2007) found that education mediated the relationship between conscientiousness (as well as agreeableness and intellect/imagination) and health behaviours, which in turn predicted health, although extraversion and agreeableness also influenced health behaviours directly, and conscientiousness showed an additional direct association with health. Later, Hampson et al. (2015) also observed that conscientiousness influenced health behaviours both directly and through educational attainment. Others have asserted that conscientiousness influences later health via educational attainment (Friedman, 2000; Jokela et al., 2013; Shanahan, Hill, Roberts, Eccles, & Friedman, 2014), and evidence of an association with education suggests that later subjective wellbeing may be influenced by personality through a similar mechanism (Witter, Okun, Stock, & Haring, 1984; DeNeve & Cooper, 1998).

Additionally, lower social class appears to predict poorer physical health (Montgomery & Carter‐Pokras, 1993; Fein, 1995; Lachman & Weaver, 1998; Hagger‐Johnson et al., 2012) and subjective wellbeing (Lachman & Weaver, 1998; Stansfield, Head, & Marmot, 1998) outcomes. Lower social class is also generally associated with lower scores on measures of traits such as extraversion and conscientiousness, and higher scores on measures of traits such as neuroticism (Eysenck & Eysenck, 1969; Lawrence & Bennett, 1992; Flensborg‐Madsen & Mortenson, 2014; Mortenson et al., 2014), as well as lower cognitive ability (Rushton & Ankney, 1996; Gottfredson, 2004; Strenze, 2007). Using their Life Course of Personality Model, Shanahan et al. (2014) suggested that socioeconomic status (SES) may be involved in the mechanistic pathway from conscientiousness, via education, to later health behaviours, health and wellbeing in different ways at different life stages. In childhood and youth, higher SES may promote higher levels of conscientiousness, and in turn (and most likely also directly) greater educational attainment. Additionally, a better education can lead to greater occupational success and thereby higher SES in later life, which affords greater access to resources, including social support, minimising stress and promoting healthier lifestyle, and in turn health and wellbeing. Furthermore, Damian, Su, Shanahan, Trautwein, and Roberts (2015) observed that the relationship between childhood SES and adult SES was mediated by conscientiousness, agreeableness and extraversion, as well as intelligence, via their effects on educational attainment. Thus, converging evidence suggests that personality, cognitive ability, education and social class are all inter‐related predictors of older‐age health and wellbeing.

### Modelling multiple predictors of health and wellbeing

However, previous studies have not generally assessed the longitudinal effects on health and wellbeing of all these factors together. Deary, Batty, Pattie, and Gale (2008) previously assessed the combined longitudinal effects of both personality and intelligence in a sample drawn from the Scottish Mental Survey 1947 (Scottish Council for Research in Education, 1949). They observed that lower IQ (assessed at around age 11) and lower dependability (a conscientiousness‐like personality dimension derived from characteristic ratings provided by teachers at around age 14) both predicted higher mortality before age 66, and that children low in both IQ and dependability were at the highest risk. We assessed the same sample, as it provided data not only on both personality and intelligence, but also on other early‐life factors, such as social class and illness. However, we focused specifically on the health of those who had survived to older age, rather than on mortality, which allowed us to assess relations to wellbeing and other contemporaneous factors that reflect quality rather than mere persistence of life. As quality of life receives increasing attention in social policy considerations regarding our ageing populations, our focus in this study is also highly relevant. Further, the sample's age, 77 years, is a time of life associated with rapid declines in health and wellbeing, and identifying lifelong influences on these is particularly important.

With this in mind, we developed a hypothetical model of the lifelong associations among personality, cognitive ability, social class and education, and their influences on older‐age health and subjective wellbeing. We expected that childhood dependability would predict older‐age health and wellbeing—as other personality traits have previously been shown to in a number of aforementioned studies—through the longitudinal stability of personality (Roberts & DelVecchio, 2000; Caspi & Roberts, 2001; Hampson & Goldberg, 2006; Edmonds, Goldberg, Hampson, & Barckley, 2013) and contemporaneous associations between personality, and health and wellbeing in older‐age (Gilhooly, Hanlon, Cullen, Macdonald, & Whyte, 2007; Gleason, Weinstein, Balsis, & Oltmanns, 2014; Weber et al., 2015). However, consistent with Hampson et al.'s (2015) argument that the cumulative consequences of lifelong health behaviours (which, once established as habits, may not require current self‐control to maintain) explain more of the association between childhood conscientiousness and older‐age health than older‐age conscientiousness does, we also hypothesised that the effects of childhood dependability operate via other pathways.

Our model focused on education as a key mediator of the influence of childhood dependability, as it has been associated with later health behaviours and health. Also, its important role as an intermediary between childhood personality and later health has already been acknowledged by Hampson et al. (2007) and, with particular reference to adolescent conscientiousness, Shanahan et al. (2014). Education may influence subsequent cognitive ability, perhaps mediating the association between cognitive ability and later health (O'Toole & Stankov, 1992; Deary et al., 2004; Hagger‐Johnson et al., 2012; Wrulich et al., 2014). Furthermore, as observed by Damian et al. (2015), we hypothesised that childhood dependability, through its influence on education (generally leading to greater career success), is associated with higher social class in adulthood, and thereby better access to resources promoting healthier lifestyle, and in turn health and wellbeing. Our hypothesised model therefore included several pathways by which childhood dependability influences older‐age health and subjective wellbeing: via routes through older‐age personality traits and education, as well as further routes through education's influence on older‐age personality, cognitive ability and social class. Both cognitive ability (Ganzach, 2000; Deary et al., 2007; Watkins, Lei, & Canivez, 2007) and social class (White, 1982; De Graaf & Huinink, 1992; Johnson, Hicks, McGue, & Iacono, 2007; Roksa & Potter, 2011) in childhood have also been observed to influence education, as well as older‐age cognitive ability (Plassman et al., 1995; Gow et al., 2011; Deary & Brett, 2015) and social class (Damian et al., 2015), respectively. We therefore modelled cognitive ability and social class in much the same way as personality, including a pathway from each childhood variable to its older‐age counterpart, as well as additional pathways via education to all older‐age variables.

### Present Study

To test our hypothesised model, we made use of data from a representative sample of Scottish children born in 1936, studied in youth from age 11 and re‐recruited at age 77. The sample provided data on personality, cognitive ability and social class in childhood and older‐age, education, and older‐age health and subjective wellbeing. A measure of childhood illness was included as an additional predictor, which we expected to influence education, as well as older‐age health and wellbeing directly. Although the sample provided data on all of these variables, it was relatively small, so our results must be considered preliminary. Nevertheless, the availability of informative data across the life course is so rare, that even a relatively small sample is of considerable value in advancing understanding of key relationships. We also attempted to verify our results in a second sample, for which data on the same older‐age variables and some of the same childhood variables were available, expecting that we could replicate the majority of our results by achieving good fit of all common aspects of the hypothesised model.

## Methods

### Participants

In this study, we focused primarily on a representative sample of Scottish people born in 1936, assessed on personality, cognitive ability and social class, both as children and as older adults, as well as lifelong education and older‐age health and wellbeing. The sample was drawn from the second Scottish Mental Survey in 1947 (SMS1947; Scottish Council for Research in Education, 1949) according to individuals' dates of birth being on one of six days of 1936—1 February, April, June, August, October and December—and thus known as the 6‐Day Sample (Macpherson, 1958). After the SMS1947, the 6‐Day Sample were studied more thoroughly, up until the age of 27 (Maxwell, 1969), during which time they were rated on dependability by their teachers, and details on social class, illness and education were recorded. In 2012, as many of the original 6‐Day Sample as possible were traced through the United Kingdom National Health Service Central Register. The 634 who were found to be still alive and resident in Scotland, England or Wales (as well as one who had emigrated) were invited to participate in a follow‐up study (Brett & Deary, 2014). Those 174 individuals (92 female; mean age 76.7 years) who agreed to take part completed a detailed questionnaire booklet, assessing personality, health and psychological well‐being and many other things. Of these participants, 131 (72 female; mean age 77.1 years) also agreed to a telephone interview, during which they completed a number of cognitive tests, referring to testing materials sent via post in advance (Deary & Brett, 2015).

To address the robustness of our observations in a larger sample, we made use of data from another sample, the 1936 Lothian Birth Cohort (LBC1936) with similar measures at similar ages. They were also participants in the SMS1947, but were not studied further at the time. They were recruited from the Community Health Index of patients living in and around Edinburgh and completed a range of follow‐up questionnaires and testing at around ages 70, 73 and 76 years of age (Deary et al., 2007; Deary, Gow, Pattie, & Starr, 2012; Lopez et al., 2012; Marioni et al., 2015). Those 866 who participated in Wave 2 in particular (448 female; mean age 72.5 years) completed many assessments that were the same or comparable to those administered to the follow‐up 6‐Day Sample in the questionnaire booklet or during the telephone interview. Further details of participants included from each study are shown in *Table* 1.

**Table 1:** Descriptive statistics for original and follow‐up samples

	SMS1947 (11 years)		6‐day sample (11 years)		LBC1936 W2 (73 years)		6DS follow‐up (77 years)	
	Mean	SD	Mean	SD	Mean	SD	Mean	SD
*N*	75 252		1208		866		174	
Males/females	38 057 / 37 195		590 / 618		448 / 418		82 / 92	
Age	10.9	0.3	10.8	0.3	72.5	0.7	76.6	0.4
Childhood MHT IQ	100.0	15.0	100.5	14.9	112.1	11.4	110.7	11.0
Years of education			10.5	1.0	10.7	1.1	11.1	1.3
Extraversion					21.6	7.2	22.3	6.7
Conscientiousness					27.7	6.1	27.9	5.5
Agreeableness					30.8	5.6	31.0	5.0
Emotional stability					25.0	7.7	26.4	6.4
Intellect/Imagination					23.7	5.9	23.8	5.9

*Note:* SMS1947 = Scottish Mental Survey of 1947; LBC1936 = Lothian Birth Cohort 1936; W2 = Wave 2; 6DS = 6‐Day Sample; MHT IQ = Moray House Test IQ. Personality was assessed in older age using a 50‐item IPIP, each scored from 0 to 4, producing a score out of 40 for each of the Big Five traits.

### Measures

#### Personality

Shortly after the SMS1947, at around 14 years of age, members of the original 6‐Day Sample were rated by their teachers on six characteristics: ‘Self‐Confidence’, ‘Perseverance’, ‘Stability of Moods’, ‘Conscientiousness’, ‘Originality’ and ‘Desire to Excel’ (MacPherson, 1958). These characteristics were selected from the longer list of traits devised by Terman (1925) for the Gifted Child Study. Teachers rated each characteristic on a five‐point scale from severely lacking to strongly displaying the characteristic. Mean childhood IQ was regressed from each of these six ratings 1Here our Methods differed slightly from those of Deary et al. (2008), as we used the mean of two measures of childhood IQ, rather than just one. This produced more clearly a single component, so we did not rotate components before extracting scores on this one. (as there were much stronger associations between childhood IQ and all characteristic ratings than are typically observed for personality characteristics). We then entered the residuals into a principal component analysis (PCA). We observed a strong single component (with loadings of .30 for Self‐Confidence, .81 for Perseverance, .54 for Stability of Moods, .81 for Conscientiousness, .52 for Originality and .77 for Desire to Excel), suggesting that the six items were closely related and measured a coherent aspect of personality. Scores on this first unrotated component were extracted, and the resulting variable was denoted Dependability, as in previous studies of the 6‐Day Sample (Deary et al., 2008; Harris, Brett, Johnson, & Deary, in revision). Consistently across the present study and previous studies, teacher‐rated childhood Dependability was most closely related to individual ratings on the Perseverance and Conscientiousness characteristics, indicating that it represents a conscientiousness‐like personality trait.

In older age, as part of the questionnaire booklet, 6‐Day Sample participants completed the 50‐item International Personality Item Pool (IPIP; Goldberg, 1999) questionnaire targeting Goldberg's (1992) markers of the Big Five factor structure (Gow et al., 2005), providing measures of Extraversion, Conscientiousness, Agreeableness, Emotional Stability (the polar opposite of Neuroticism) and Intellect/Imagination (similar to Openness). Participants also rated themselves in older age on the same six characteristics that their teachers rated them on in childhood. We derived a measure of older‐age Dependability from a PCA of these self‐ratings. We chose to use the more widely validated IPIP scales as measures of older‐age personality in our models, but we computed correlations with these and other important study variables for older‐age Dependability. Dependability was not assessed in the LBC1936, but their older‐age personality was also assessed using the same IPIP questionnaire at Wave 2 testing.

#### Cognitive ability

Almost all children born in 1936 and present at school in Scotland on the day of the SMS1947 completed the Moray House Test No. 12, an IQ‐type test of general intelligence (Scottish Council for Research in Education, 1933, 1949). Scores on the test were later converted to IQ‐type scores by residualising over age and standardising to the typical IQ scale (M = 100.0, SD = 15.0). Every 6‐Day Sample participant (including those 96 who were absent on the day of the SMS1947) also completed Terman & Merrill's, 1937 revision of the Stanford–Binet IQ test, administered individually (Scottish Council for Research in Education, 1949). Childhood IQ was represented by the mean of the two IQ scores in the 6‐Day Sample (for those who did not complete the Moray House Test, we used Stanford–Binet IQ alone; the correlation between the two was .81). In older‐age, the 6‐Day Sample follow‐up participants who received the telephone interview completed Raven's Standard Progressive Matrices (Raven, 1938), the Rey Auditory Verbal Learning Test (Rey, 1964; Schmidt, 1996), the Symbol Digit Modalities Test (Smith, 1982) and a short test of semantic fluency, which involved naming as many animals as possible in 1 min. These tests were administered using materials sent to the participants in advance. Testing materials were sent by post in a sealed envelope, which participants were instructed to leave sealed until the time of the interview (Brett & Deary, 2014; Deary & Brett, 2015). Similar tests of matrix reasoning (from the third edition of the Wechsler Adult Intelligence Scale; WAIS‐III; Wechsler, 1997), verbal memory (from the accompanying Wechsler Memory Scale; WMS‐III; Psychological Corporation, 1997), symbol decoding (also from the WAIS‐III) and verbal fluency were administered in person to LBC1936 participants at Wave 2 testing. Scores on these four tests were standardised and reduced to a single dimension representing older‐age Cognitive Ability using a two‐group confirmatory factor analysis (CFA), as described below.

#### Social class

In the 6‐Day Sample questionnaire booklet and in Wave 1 of the LBC1936 follow‐up, participants reported their highest‐level occupation. Adult Social Class was derived from this information, coded according to the 1980 UK census classification of occupations (Stevens & Cho, 1985). The occupations of 6‐Day Sample participants' fathers were also recorded in 1947 (Scottish Council for Research in Education, 1949), while those of LBC1936 participants' fathers were recorded retrospectively, providing a measure of childhood Social Class, coded according to the 1951 UK census classification of occupations (General Register Office, 1956).

#### Education

Details on 6‐Day Sample participants' education and attainment were recorded during the annual follow‐up assessments to age 27, from which we derived two variables: years of full‐time education and level of highest qualification, ranked from 0 (no qualifications) to 5 (university degree). LBC1936 participants reported the same information retrospectively in older age. For each sample, the mean of the two standardised variables (showing a correlation of .54) was used as an overall measure of Education.

#### Health

In older‐age, both samples were asked about a range of specific current health conditions: cancers, cardiovascular diseases, diabetes, high blood pressure, high cholesterol, circulatory problems, arthritis, hyper‐ or hypothyroidism, Parkinson's disease and dementia. Details of previous cancers and strokes were also reported. The total number of current conditions (and previous incidences of cancer or stroke) reported, reversed to produce a score that was higher for individuals with fewer health conditions, served as one self‐report measure of health in older‐age (c.f. Johnson & Krueger, 2005a, 2005b). All participants also provided details of any medications they were currently taking, and the total number was reversed and used as a second self‐report measure of health. The 6‐Day Sample completed the Medical Outcomes Study 36‐item Short‐Form Health Survey (SF‐36; Ware & Sherbourne, 1992; McHorney, Ware, & Raczek, 1993) as part of the questionnaire booklet, and the total score on the physical functioning and physical role functioning subscales was used as a self‐rated measure of health‐related functional ability. In the LBC1936, health‐related functional impairment was self‐rated at Wave 2 in terms of activities of daily living (ADL), using the Townsend Functional Ability Scale (Townsend, 1962). These three measures were standardised and reduced to a single dimension, representing older‐age Health, using a constrained two‐group CFA, as for Cognitive Ability. In the 6‐Day Sample, serious health conditions were recorded at around 14, 15, 17 and 18 years of age (MacPherson, 1958). A record of serious illness at any of these times was used as a simple binary measure of Childhood Illness.

#### Subjective wellbeing

As part of the 6‐Day Sample questionnaire booklet, and in person at LBC1936 Wave 2 testing, all participants completed the 14‐item Warwick–Edinburgh Mental Well‐Being Scale (Tennant et al., 2007), the 5‐item Satisfaction With Life Scale (Diener, Emmons, Larsen, & Griffin, 1985) and the 10‐item revised Life Orientation Test (Scheier, Carver, & Bridges, 1994). As for Cognitive Ability and Health, self‐ratings on these three measures of subjective wellbeing were standardised and reduced to a single dimension representing older‐age Wellbeing using a constrained two‐group CFA.

### Analysis

Data were analysed in SPSS 19 (IBM, Armonk, New York) and modelled using the accompanying AMOS package. Individual measures were first square or square‐root transformed, if substantially positively or negatively skewed, and then standardised. Outlying data points were capped at three SDs from the mean, and any variables missing individual data points were completed by maximum likelihood estimation. However, participants missing data for multiple key variables—three 6‐Day Sample participants and 12 LBC1936 participants who had not completed the personality and wellbeing questionnaires at follow‐up, and a further eight LBC1936 participants for whom no childhood data were available—were excluded from further analyses. Data on childhood IQ and Education were reduced by calculating the mean of the two standardised measures of each. As above, the six teacher ratings of adolescent characteristics were regressed over childhood IQ and then submitted to PCA. Scores on the first unrotated component were taken as a measure of childhood Dependability. For older‐age cognitive ability, scores on the four cognitive tests administered during the follow‐up studies were reduced to a single underlying factor using CFA. The four tests were modelled as indicators of a single latent variable, and all parameters were constrained equal across the two samples. Calculated scores on the derived latent variable were used as the measure of older‐age Cognitive Ability. Number of self‐reported health conditions, self‐reported medications and self‐rated functional ability were reduced to a single dimension representing older‐age Health by the same method, as were the three questionnaires measuring aspects of older‐age Wellbeing. Relations among the averaged and reduced measures, as well as other single‐measure variables, were initially assessed by calculating Pearson's correlation coefficient for each pair of variables. Older‐age Dependability was also included in these analyses.

We then entered our 14 variables of interest into structural equation models of older‐age Health and Wellbeing in 6‐Day Sample follow‐up participants. These two outcomes were first modelled separately, as functions of childhood Dependability, IQ, Social Class and Illness, via older‐age personality traits, Cognitive Ability and Social Class. We also tested alternative models of older‐age Health and Wellbeing, each additionally including the other as an intermediary variable. After running each initial hypothesised model, it was constrained by fixing the regression weights of non‐significant paths at zero—one at a time, and re‐running the model after each change—until all free paths showed significant effects at *p* < .05. In our next step, we excluded the constrained non‐significant paths from the model, along with any variables that were no longer linked to any other variables. We also tested a further‐reduced model, which additionally excluded any variables that did not have either a significant direct path to the primary outcome variable, or a significant path to an intermediary variable with a significant path to the outcome variable. Although sex may have played roles in some of the relations we assessed, we are not aware of theories specifying such relations and sample size did not provide enough power to model data for males and females separately. All models thus included males and females together.

To test the generalisability of our results to another and larger sample, we repeated this modelling process, first excluding elements of the initial model that were unique to the 6‐Day Sample. Finally, we ran two‐group constrained models of older‐age Health and Wellbeing, constraining measurement weights, intercepts, residuals and structural covariances equal across the two samples, but also constraining path coefficients to those estimated in the models of 6‐Day Sample data, including only variables common to both samples. Because both the full and constrained models offered meaningful information, we present results in a format reflecting all of them.

## Results

Descriptive statistics for participants in the two samples are reported in *Table* 1. The original 6‐Day Sample participants were very similar to the entire SMS1947 cohort in childhood IQ, but, as reported previously (Johnson, Brett, Calvin, & Deary, 2016), surviving through to older age and agreeing to participate in the follow‐up study was not random. Those who were able and willing to participate in the LBC1936 and 6‐Day Sample follow‐up studies in older‐age had substantially higher than average IQs as children (the cognitive ability differences between each of these two follow‐up samples and the full cohort had Cohen's d effect sizes of .78 and .81, respectively). The 6‐Day Sample follow‐up participants had remained in education longer than those in LBC1936, by just under six months on average (d = .35), which was likely because of greater sample selectivity at their later age of recruitment and the greater cognitive demands imposed by self‐administration of the study tasks. Personality trait scores in older age were similar for the two follow‐up samples. The results of the CFAs, used to support creation of latent representations of older‐age Cognitive Ability, Health and Wellbeing as outcome variables, are reported in *Table* 2. Reduction to the same single dimension across samples was appropriate for each outcome, with each two‐group model showing good fit (Cognitive Ability: CFI = .954, RMSEA = .071; Health: CFI > .999, RMSEA = .013; Wellbeing: CFI > .999, RMSEA = .011). Constraining factor loadings, covariances and residuals equal across samples did not result in a substantial loss of fit for any of the three two‐group models (Cognitive Ability: CFI = .943, RMSEA = .046; Health: CFI = .994, RMSEA = .017; Wellbeing: CFI > .999, RMSEA < .001). In each case, scores on the latent variable in the fully constrained model were taken as the measure of each construct.

**Table 2:** Confirmatory factor analysis results

Latent variable	Indicator variable	Standardised estimate	Standard error
Older‐age Cognitive Ability	Matrix reasoning	.534	
	Symbol‐digit translation	.668	.122
	Semantic fluency	.516	.097
	Verbal memory	.490	.098
Older‐age Wellbeing	WEMWBS	.764	
	Satisfaction with life	.651	.032
	Optimism	.752	.031
Older‐age Health	Self‐reported conditions	.724	
	Self‐reported medications	.696	.095
	Self‐rated functional impairment	.469	.059

*Note:* WEMWBS = Warwick–Edinburgh Mental Well‐Being Scale. Confirmatory factor analyses were performed for the three latent variables with more than two indicators, extracting a single underlying factor, while constraining measurement weights, intercepts and residuals, and structural covariances equal across samples.

Correlations among these latent variables and other measures are reported in *Table* 3. In the 6‐Day Sample, older‐age Health showed a moderate negative correlation with childhood Illness, and weak to moderate positive correlations with childhood IQ, Education, adult Social Class, and older‐age Extraversion, Emotional Stability, Intellect/Imagination, Cognitive Ability and Wellbeing. These correlations were similar in the LBC1936, except that the correlations with Wellbeing and Social Class were weaker, but still significant, and there was no correlation with older‐age Extraversion. Wellbeing showed relatively strong positive correlations with all other older‐age variables in the 6‐Day Sample, especially Emotional Stability, as well as weak to moderate positive correlations with childhood IQ, Education and adult Social Class, and a weak negative correlation with childhood Illness. Again, these correlations were similar in the LBC1936, although that with older‐age Cognitive Ability was weaker. Although not included in the models, correlations between older‐age Dependability and all other variables are included in *Table* 3. Older‐age Dependability was moderately related to older‐age Conscientiousness (.48), but did show similar correlations with the other four personality scales, particularly Extraversion (.44) and Intellect/Imagination (.44), as well as with Wellbeing (.53). All these measures showed substantial inter‐correlations, which has also been observed in many other studies.

**Table 3:** Correlations among all variables

		1	2	3	4	5	6	7	8	9	10	11	12	13	14	15
1	Childhood IQ	—	.02	**.22**	**−.10**	**.49**	**.35**	.13	**.20**	.13	−.08	**.28**	**.33**	**.52**	.12	**.18**
2	CH Dependability		—	**.09**	−.05	**.18**	.10	−.06	−.06	.04	−.14	.10	−.03	−.04	−.03	−.04
3	CH Social Class	**.20**		—	−.05	**.29**	**.21**	−.05	.02	−.15	.05	.03	.06	.12	−.08	−.02
4	CH Illness				—	−.05	−.04	−.01	−.02	.13	−**.18**	−.13	.05	−.10	−.11	−**.19**
5	Education	**.49**		**.35**		—	**.46**	.14	.05	.08	−.02	**.25**	**.40**	**.44**	.13	**.19**
6	Adult Social Class	**.39**		**.23**		**.55**	—	.11	.11	.07	.05	**.16**	.15	**.33**	.11	**.18**
7	Older‐age Dependability							—	**.44**	**.20**	**.48**	**.29**	**.44**	**.22**	**.53**	.08
8	OA Extraversion	**.09**		−.02		**.11**	**.12**		—	**.29**	**.15**	**.24**	**.37**	.15	**.34**	.12
9	OA Agreeableness	.06		.06		.01	**.09**		**.31**	—	**.31**	**.21**	**.25**	.11	**.35**	−.09
10	OA Conscientiousness	.00		−.03		.03	.05		**.13**	**.29**	—	**.18**	**.20**	**.21**	**.43**	.08
11	OA Emotional Stability	**.12**		.02		**.09**	**.11**		**.23**	**.19**	**.24**	—	**.25**	**.25**	**.51**	**.24**
12	OA Intellect/Imagination	**.29**		.10		**.34**	**.27**		**.42**	**.31**	**.18**	**.16**	—	**.31**	**.24**	.14
13	OA Cognitive Ability	**.55**		**.15**		**.45**	**.36**		**.09**	**.11**	.06	**.14**	**.31**	—	**.33**	.16
14	OA Wellbeing	**.16**		.01		**.16**	**.16**		**.35**	**.34**	**.34**	**.59**	**.27**	**.18**	—	**.36**
15	OA Health	**.12**		−.08		**.15**	**.07**		.01	.01	**.10**	**.13**	.04	**.19**	**.14**	—

*Note:* CH = childhood; OA = older‐age; 1 = mean of two measures of childhood IQ; 2 and 7 = scores on first unrotated component derived by principal component analysis; 5 = mean of two measures of education; 12–14 = scores on single factor derived by each confirmatory factor analysis. Pearson's correlation coefficients for associations among observed and latent variables in the 6‐Day Sample are included above the diagonal, and for the same associations in the 1936 Lothian Birth Cohort below. Significant correlations at *p* < .05 (with no adjustment for multiple testing) are highlighted in bold.

**Figure 1:**
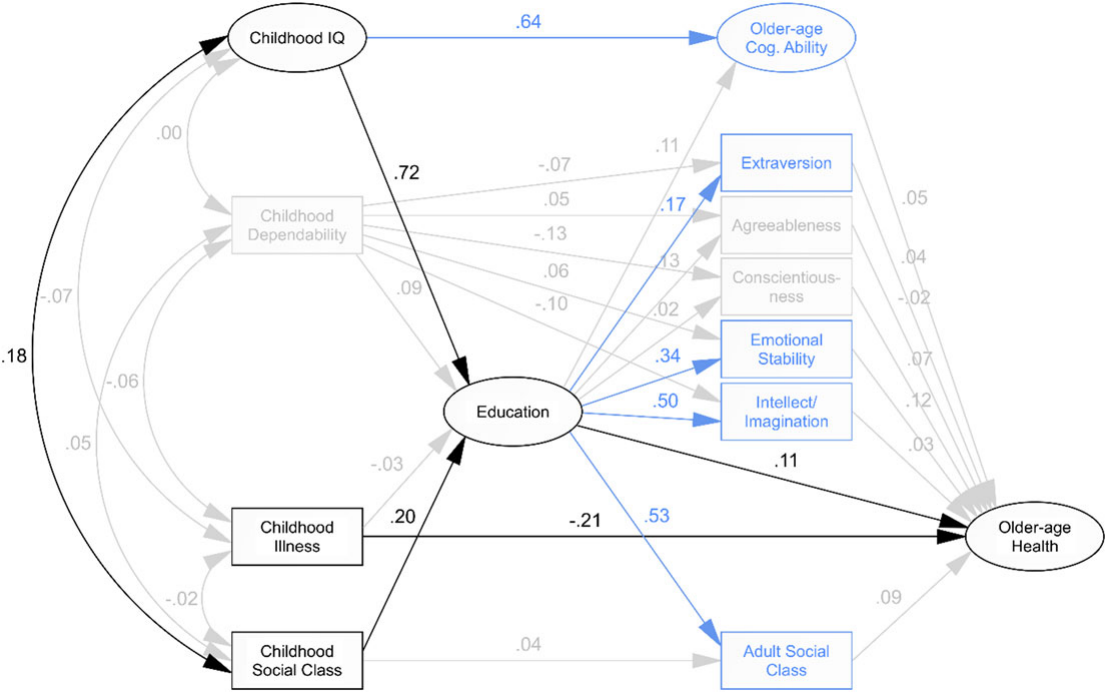
*Model of older‐age Health*. Paths shown in grey were non‐significant at *p* < .05 and were therefore constrained to zero in the constrained model and removed from subsequent models. Variables in grey showed no significant association with any other variables and were therefore removed from the trimmed and final models. Variables shown in blue had no direct or indirect effect on the primary outcome, older‐age Health, and were therefore removed from the final model. Path labels represent standardised regression weights derived from the illustrated initial model, which, for retained paths, did not differ substantially in subsequent models.

Our initial hypothesised model of older‐age Health, illustrated in *Figure* 1, fit the 6‐Day Sample data reasonably well (CFI = .835, RMSEA = .073; *Table* 4). Parameter estimates indicated significant effects of childhood IQ and Social Class on older‐age Health via Education, as well as a direct effect of childhood Illness. However, the regression weights estimated for many of the paths—for example, those between childhood Dependability and later‐life variables—indicated no significant effect. Constraining these coefficients to zero did not result in a substantial change in other path coefficients, nor a significant loss in model fit (CFI = .834, RMSEA = .069). With these paths constrained to zero, childhood Dependability, and older‐age Agreeableness and Conscientiousness were no longer linked to any other variables in the model. After removing all constrained paths, along with these subsequently isolated variables, all other path coefficients remained the same, and again, there was no significant loss in model fit (CFI = .911, RMSEA = .058). This trimmed model indicated that older‐age Cognitive Ability was related to childhood IQ—as shown previously in the 6‐Day Sample (Deary & Brett, 2015) and LBC1936 (Gow et al., 2011)—and that older‐age Extraversion, Emotional Stability, Intellect/Imagination and Social Class were related to early‐life variables via education; yet none of these later‐life factors were in turn associated with older‐age Health. Our final step was to remove these variables as well. Again, this did not result in a substantial change in the coefficients of other paths, and actually produced an improvement in overall model fit (CFI = .992, RMSEA = .028).

**Table 4:** Fit statistics for all models of 6‐Day Sample data

Model	χ^2^	df	p	χ^2^/df	NFI	CFI	RMSEA
*Older‐age Health*							
Initial	287.43	149	<.001	1.93	.726	.835	.073
Constrained	310.26	171	<.001	1.81	.704	.834	.069
Trimmed	185.87	117	<.001	1.59	.799	.911	.058
Final	29.61	26	.284	1.14	.943	.992	.028
*Older‐age Wellbeing*							
Initial	307.62	149	<.001	2.07	.737	.835	.079
Constrained	326.80	170	<.001	1.92	.721	.837	.073
Trimmed	277.03	133	<.001	2.08	.753	.848	.079
Final	125.90	53	<.001	2.38	.830	.890	.089
*Older‐age Health via Wellbeing*							
Initial	473.23	208	<.001	2.28	.660	.762	.086
Constrained	496.08	230	<.001	2.16	.643	.761	.082
Trimmed	369.56	167	<.001	2.21	.708	.808	.084
Final	109.38	52	<.001	2.10	.855	.915	.080
*Older‐age Wellbeing via Health*							
Initial	393.12	208	<.001	1.89	.717	.834	.072
Constrained	408.63	226	<.001	1.81	.706	.836	.069
Trimmed	380.83	204	<.001	1.87	.721	.841	.071
Final	202.64	100	<.001	2.03	.787	.874	.077

*Note:* Initial = full initial hypothesised model of 6‐Day Sample data; Constrained = non‐significant paths constrained to zero; Trimmed = non‐significant paths and isolated variables removed; Final = variables with no direct/indirect effect on the primary outcome also removed.

**Figure 2:**
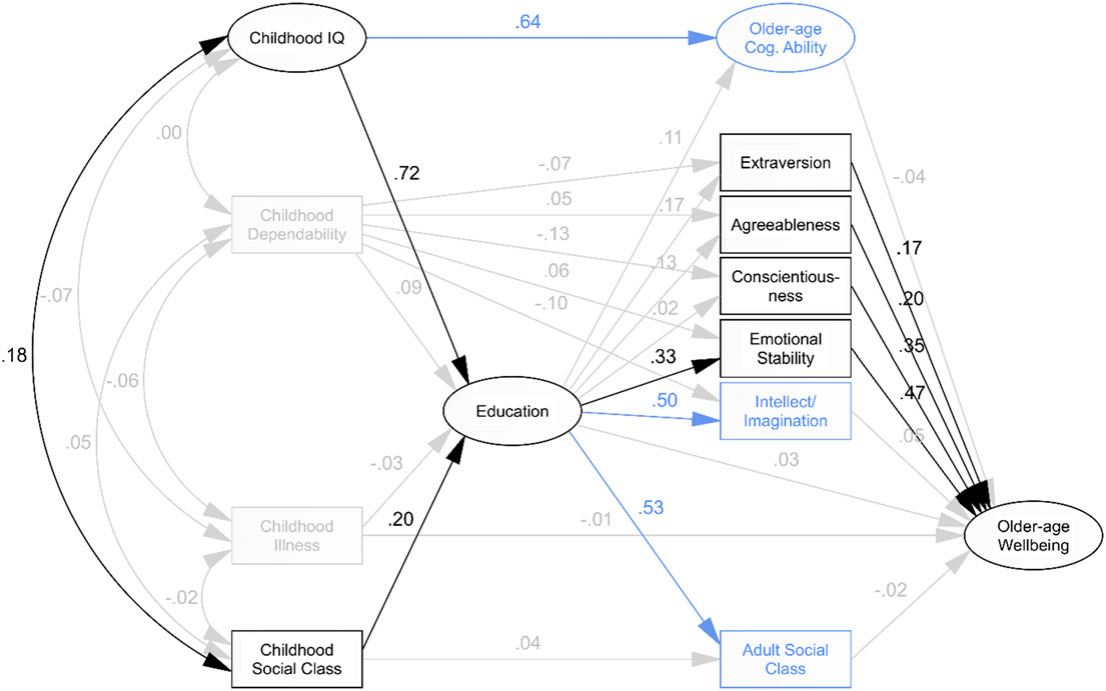
*Model of older‐age Wellbeing*. Paths shown in grey were non‐significant at *p* < .05 and were therefore constrained to zero in the constrained model and removed from subsequent models. Variables in grey showed no significant association with any other variables and were therefore removed from the trimmed and final models. Variables shown in blue had no direct or indirect effect on the primary outcome, older‐age Health and were therefore removed from the final model. Path labels represent standardised regression weights derived from the illustrated initial model, which, for retained paths, did not differ substantially in subsequent models.

We modelled older‐age Wellbeing in exactly the same way as older‐age Health (*Figure* 2). Again, the initial model fitted reasonably well (CFI = .835, RMSEA = .079; *Table* 4), but many estimated path coefficients suggested no significant effect. Constraining non‐significant paths to zero produced no loss of model fit (CFI = .837, RMSEA = .073) and no substantial change to other parameters. Again, childhood Dependability and, this time, childhood Illness were left with no link to any other variables in the model. When non‐significant paths, childhood Dependability and childhood Illness were removed from the model, path coefficients remained unchanged, and there was still no significant loss of model fit (CFI = .848, RMSEA = .079). As for older‐age Health, the trimmed model still included variables with no effect on the primary outcome, although this time only three: older‐age Cognitive Ability, Intellect/Imagination and Social Class. Removing these variables produced no notable change in model fit (CFI = .890, RMSEA = .089) or other path coefficients.

**Figure 3:**
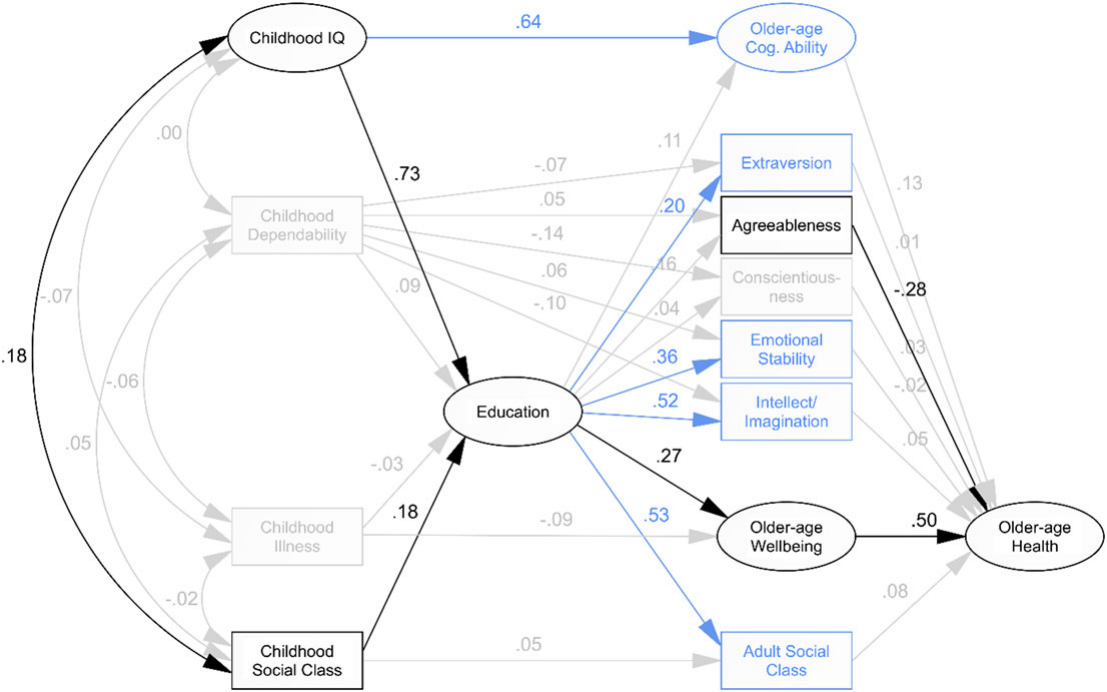
*Model of older‐age Health via older‐age Wellbeing*. Paths shown in grey were non‐significant at *p* < .05 and were therefore constrained to zero in the constrained model and removed from subsequent models. Variables in grey showed no significant association with any other variables and were therefore removed from the trimmed and final models. Variables shown in blue had no direct or indirect effect on the primary outcome, older‐age Health, and were therefore removed from the final model. Path labels represent standardised regression weights derived from the illustrated initial model, which, for retained paths, did not differ substantially in subsequent models.

An alternative hypothesised model of older‐age Health incorporated older‐age Wellbeing as an intermediary variable (between childhood Illness and Education, and older‐age Health; *Figure* 3). In contrast to the model of older‐age Health without Wellbeing, this model estimated a significant association between older‐age Agreeableness and Health, indicated no association with childhood Illness, and did not fit as well (CFI = .762, RMSEA = .086; *Table* 4). Constraining non‐significant paths (CFI = .761, RMSEA = .082) or removing these paths, along with childhood Dependability and Illness, and older‐age conscientiousness (CFI = .808, RMSEA = .084) made little difference to either path coefficients or model fit. The same older‐age variables—Cognitive Ability, Extraversion, Emotional Stability, Intellect/Imagination and Social Class—were significantly associated with early‐life variables, but not with older‐age Health. Again, these variables could be removed without affecting other model parameters (CFI = .915, RMSEA = .080).

**Figure 4:**
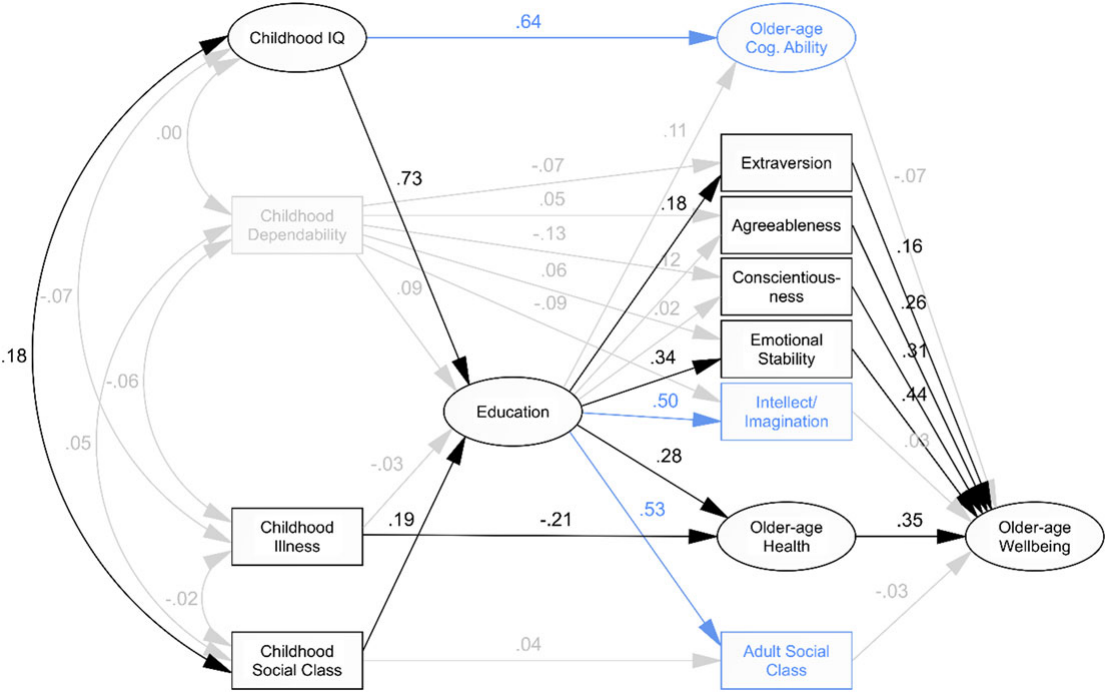
*Model of older‐age Wellbeing via older‐age Health*. Paths shown in grey were non‐significant at *p* < .05 and were therefore constrained to zero in the constrained model and removed from subsequent models. Variables in grey showed no significant association with any other variables and were therefore removed from the trimmed and final models. Variables shown in blue had no direct or indirect effect on the primary outcome, older‐age Health, and were therefore removed from the final model. Path labels represent standardised regression weights derived from the illustrated initial model, which, for retained paths, did not differ substantially in subsequent models.

Our fourth and final way of modelling data from the 6‐Day Sample included older‐age Wellbeing as the primary outcome, with older‐age Health as an intermediary variable (*Figure* 4). This model did fit reasonably well (CFI = .834, RMSEA = .072), and estimated non‐significant coefficients for fewer paths than any of the previous models. Constraining these to zero did not affect model fit (CFI = .836, RMSEA = .069) and left only childhood Dependability isolated from the rest of the model. Model fit and other path coefficients remained about the same when non‐significant paths and childhood Dependability were removed (CFI = .841, RMSEA = .071) and when further variables with no effect on older‐age Wellbeing—older‐age Cognitive Ability, Intellect/Imagination and Social Class—were also removed (CFI = .874, RMSEA = .077).

We tested alternative ways of modelling particular variables to evaluate sensitivity of results to our measurement choices. First, we repeated the four models described above using childhood Dependability without regressing mean childhood IQ from the items. This made slight differences to the models (most notably, the unexpectedly negative association between childhood Dependability and older‐age Conscientiousness was marginally significant, although not significantly stronger, in each model), but the effects of childhood Dependability and IQ on intermediary variables with significant paths to older‐age Health and Wellbeing did not change substantially.

We also repeated each of the three models including older‐age Health, but without including self‐rated functional ability as an indicator, it being less closely related to the other Health indicator variables. Again, this had only small effects on the models (for example, in the simpler model of Health, the direct effect of Education was slightly smaller, while its indirect effect via adult Social Class was slightly greater), and overall the results were not substantially different. Finally, in order to test whether the small (and, in most cases, non‐significant) effects of childhood Dependability we observed were because of their being suppressed by the greater influences of other early‐life factors, we repeated the four models without including childhood IQ, Illness and Social Class. The coefficients of all paths from childhood Dependability remained small and non‐significant in each case.

To test the robustness of these results, we investigated whether the estimated parameters could also be fitted to data from the larger LBC1936 sample. However, data on childhood Dependability and Illness were unavailable there. We therefore first repeated the above models of 6‐Day Sample data, but excluding the childhood Dependability and Illness variables. As childhood Dependability was not retained in any of the final 6‐Day Sample models, its exclusion made no difference. For the two models that included a significant direct effect of childhood Illness on older‐age Health, excluding childhood Illness did not substantially affect model fit, but it did mean the regression weights were slightly higher for paths from childhood IQ and Social Class to Education, and from Education to older‐age Health.

Each of the four initial joint models fit data from both groups together relatively well (Health: CFI = .813, RMSEA = .056; Wellbeing: CFI = .833, RMSEA = .059; Health via Wellbeing: CFI = .728, RMSEA = .067; Wellbeing via Health: CFI = .834, RMSEA = .052; *Table* 5). Constraining all weights, intercepts, covariances and residuals equal across the two samples produced no substantial loss in fit of any of the four models (Health: CFI = .821, RMSEA = .048; Wellbeing: CFI = .839, RMSEA = .051; Health via Wellbeing: CFI = .735, RMSEA = .059; Wellbeing via Health: CFI = .840, RMSEA = .046). Finally, we constrained the parameters of the two‐group models to those estimated at each step of reducing the models of 6‐Day Sample data, and even when constrained to the parameters of the final 6‐Day Sample models, still observed no notable loss in model fit (Health: CFI = .936, RMSEA = .034; Wellbeing: CFI = .842, RMSEA = .059; Health via Wellbeing: CFI = .887, RMSEA = .047; Wellbeing via Health: CFI = .837, RMSEA = .052). Our main results, although derived from the relatively small 6‐Day Sample, did therefore appear to be reasonably robust.

**Table 5:** Fit statistics for all two‐group models

Model	χ^2^	df	p	χ^2^/df	NFI	CFI	RMSEA
*Older‐age Health*							
Free	868.97	216	<.001	4.02	.771	.813	.056
Constrained	175.73	87	<.001	2.02	.881	.936	.034
*Older‐age Wellbeing*							
Free	961.15	216	<.001	4.45	.798	.833	.059
Constrained	557.71	128	<.001	4.36	.804	.842	.059
*Older‐age Health via Wellbeing*							
Free	1686.08	316	<.001	5.34	.691	.728	.059
Constrained	377.16	125	<.001	3.02	.841	.887	.047
*Older‐age Wellbeing via Health*							
Free	1151.60	316	<.001	3.64	.789	.834	.052
Constrained	692.41	203	<.001	3.41	.785	.837	.052

*Note:* Free = full initial hypothesised model applied to data from both groups (6‐Day Sample and Lothian Birth Cohort 1936) together, without constraining any parameters; Constrained = final two‐group model, constraining regression weights to those from the final model of 6‐Day Sample data (excluding childhood Dependability and Illness) and constraining all other parameters equal.

## Discussion

In this study, we assessed the lifelong associations among personality, cognitive ability, social class and education, and their influence on health and subjective wellbeing in older‐age. We modelled older‐age Health and Wellbeing as separate outcomes, and each as a possible mediator of the other. Our various models estimated some effects of personality that we had expected, although often weaker than expected, and some associations that we had not anticipated. For instance, the separate model of older‐age Health indicated that the influence of childhood Dependability was small, showing positive associations with Education and older‐age Agreeableness and Emotional Stability, and unexpectedly negative associations with older‐age Extraversion, Conscientiousness and Intellect/Imagination. However, none of these associations was significant or included in the final reduced model. Older‐age personality traits also showed no significant associations with older‐age Health, the strongest being with Emotional Stability at .12. However, when Wellbeing was included as an intermediary variable, older‐age Agreeableness showed a significant negative association of −.28 with older‐age Health. This suggested that multicollinearity among the personality scales may have partly obscured their effects on Health. That Agreeableness emerged this way as negatively associated with Health could indicate that inclusion of Wellbeing released expression of the part of the assertive aspects of lack of agreeableness that have sometimes been related to better health outcomes, or it could indicate that the variance common to Wellbeing and the personality scales in general created a spurious association between Agreeableness and Health. More detailed measures over longitudinal time frames would be needed to distinguish these possibilities. In the separate model of Wellbeing, the effects of childhood Dependability were similarly small and non‐significant, but older‐age Extraversion, Agreeableness, Conscientiousness and Emotional Stability each showed significant small to moderate associations with Wellbeing, suggesting that personality was much more closely linked to Wellbeing than Health. In support of this, including older‐age Health as an intermediary made little difference to any of the paths from childhood or older‐age personality variables.

In these samples, Education influenced older‐age Health and Wellbeing, acting as a mediator of other early‐life influences. Childhood IQ, for example, was a significant predictor of Health and Wellbeing in each of the four models, but via Education, rather than via older‐age cognitive ability. The effects of older‐age Cognitive Ability on Health and Wellbeing were close to zero and not significant. Similarly, childhood Social Class contributed significantly to each outcome, but via Education, rather than via adult Social Class, whose effects on Health and Wellbeing were also close to zero and not significant. Childhood Illness showed significant effects of .21 and .23 on older‐age Health in the two models that included this direct path, but showed only small associations of −.01 and −.09 with Wellbeing in the other two models, which were not significant. This provided some indication that Health was more important to Wellbeing than Wellbeing was to Health. Better fit of the final models of Health and Wellbeing via Health than those of Wellbeing and Health via Wellbeing supported this idea. Finally, we attempted to replicate our results in a larger sample. Each of the four models was fitted to data from both samples, constraining path coefficients to those estimated for the 6‐Day Sample without any substantial loss of fit. This provided some evidence that the associations among variables that we observed in this study may extend to a wider population.

### Study Limitations

Before discussing the implications of our results, we must consider several important limitations of this study. First, our exploratory sample, the 6‐Day Sample, was rather small. Although the original 6‐Day Sample included 1208 participants in 1947, only 174 of these individuals participated in the follow‐up study (of which 131 completed cognitive testing at the final stage of the study). In some instances where we observed no association between variables, there may actually have been associations that were not strong enough to be detected in this small sample. Our results must therefore be considered preliminary, but still should be considered important, because of the overall scarcity of longitudinal data spanning this wide swatch of the lifespan that links childhood conditions and characteristics with those in late life. Furthermore, we addressed this limitation by replicating our results in a second sample that offered common late‐life data, one important common childhood variable, and similar though retrospective reports of several other variables. This LBC1936 was much larger, but it did lack the key childhood measures of Dependability and Illness. This could have emerged as a more important limitation than it appears to have, however, as the estimated contribution of childhood Dependability to each of the 6‐Day Sample models of older‐age Health and Wellbeing was small, and excluding childhood Illness made little difference to the final models in which it featured. The fact that the estimated models fit data from both groups similarly well therefore suggests that the associations we observed in the 6‐Day Sample are likely present in larger samples and similar more general populations.

Both samples, however, were subject to a related limitation: those who participated generally had higher IQs as children than the SMS1947 population from which they were drawn (Gow et al., 2011; Deary & Brett, 2015), and this was also true of the Dependability ratings of the 6‐Day Sample follow‐up participants (Johnson et al., 2016). These differences were because of both childhood IQ and dependability being associated with surviving into older age (Deary et al., 2008), but much more strongly, among those who were alive and traced in older age, with volunteering to participate in the follow‐up study. Although similar biases likely affect any sample studied from childhood to older age, the restriction in range introduced by these biases in the current study's two samples further reduced the reliability of the estimated models parameters. Also because of the small sample size, we were unable to investigate sex differences by modelling data from males and females separately. This should be considered in future studies using larger samples.

Additionally, there were several weaknesses in the childhood Dependability measure used in the 6‐Day Sample. First, although the characteristics rated were developed through previous research (Terman, 1925), they were not rooted in any surviving major theory or model of personality. However, the underlying component that could be derived from these ratings, Dependability, can conceptually be closely associated with conscientiousness and other similar traits that do feature in some of the current models of personality. The 6‐Day Sample has also been assessed on Dependability in older‐age and showed associations with each of the Big Five personality traits, but a particularly strong correlation with Conscientiousness (Harris et al., in revision). Still, this highlights that the characteristic ratings used did not assess the full breadth of personality measured by the questionnaires that have since become available.

Second, the six characteristic ratings were single‐item measures, which may not have been particularly reliable, although we only used their underlying component, Dependability, thereby essentially creating a six‐item measure. Third, the six characteristics assessed in adolescence were rated by participants' teachers, who tended to observe the participants only in the strongly achievement‐oriented school setting and were also responsible for rating the quality of the Dependability and cognitive ability‐eliciting tasks they assigned the participants to perform. We believe that these circumstances may have caused teachers' assessments of ability to impact their Dependability ratings, as evidenced by substantial (considerably greater than typical personality–IQ) correlations with childhood IQ scores, so we residualised mean childhood IQ from the individual item ratings before reducing them to a single dimension. However, this would not have accounted for other sources of bias and could have removed purely Dependability‐related variance (Deary & Johnson, 2010). We therefore repeated our models using an alternative measure of Dependability derived from the non‐residualised teacher ratings, which made little difference to results. While some evidence suggests that teacher ratings provide reliable estimates of personality (Baker, Victor, Chambers, & Halverson, 2004; Pulkkinen, Kokko, & Rantanen, 2012), others have found that they are least closely related to those of other raters (Laidra, Allik, Harro, Merenäkk, & Harro, 2006). This could attest to the constraints on behaviour imposed by the circumstances of the school environment, but it may also attest to effects of differences in norms of behaviour through which to assess relative personality characteristics in people's family and friendship networks from which self‐ and other‐raters are usually drawn.

A further limitation of this study lies in investigating the relationship between personality and subjective wellbeing, as these two constructs seem to overlap, each having a theoretical basis in affect (Schmutte & Ryff, 1997). The two constructs are also measured using self‐ratings of some remarkably similar items; for example, the Warwick–Edinburgh Mental Well‐Being Scale includes items on cheerfulness and interest in others, which are also used by the 50‐item IPIP questionnaire to measure emotional stability and extraversion, respectively. Schmutte and Ryff suggested that the observed correlations between personality traits and subjective wellbeing may be largely attributable to this overlap of constructs. It is therefore unclear how meaningful any associations between personality traits and subjective wellbeing are, or whether it is reasonable to consider, for instance, distinct effects of personality and subjective wellbeing on health. Finally, not only were older‐age personality and wellbeing self‐rated, but the three measures of health were also based on self‐reports. We discuss our results below within the context of these limitations.

### Personality associations

One of the main findings of this study was that childhood Dependability did not contribute significantly to older‐age Health or Wellbeing. This finding stands in contrast to the results of previous studies indicating a relationship between childhood personality and health and wellbeing in later life (Friedman, 2000; Hampson et al., 2007; Friedman et al., 2010; Bogg & Roberts, 2013; Hampson et al., 2015). More specifically, we hypothesised that Dependability would influence older‐age Health and Wellbeing via older‐age personality traits, but none of the models included significant paths from childhood Dependability to any older‐age personality traits. This seems inconsistent with previous evidence of personality stability over many decades (Roberts & DelVecchio, 2000; Caspi & Roberts, 2001; Hampson & Goldberg, 2006; Edmonds et al., 2013). However, we have previously assessed relations among the 6‐Day Sample between dependability as rated by teachers in adolescence, and as rated by participants and their friends or family members in older age. We observed only weak correlations that did not achieve significance, suggesting that stability may be lower over more than six decades (Harris et al., in revision). Alternatively, the low stability of Dependability could be because of the very different life circumstances in which the teachers in childhood, and self‐ and other‐raters (usually spouses or other close relatives such as offspring) in old age observed the participants on the highly achievement‐oriented items. These items assessed behaviours elicited daily in the childhood circumstances in which the teachers observed them, but rarely in old age. Further, as above, the sample was not sufficiently powered to detect an effect of the size that might be expected for personality stability over nearly seven decades, and significant effects of childhood Dependability on older‐age Health and Wellbeing may have been observed in the present study had the sample been larger.

We also predicted an influence of childhood Dependability via Education. There was a small positive association between Dependability and Education in each model, but this was not strong enough to be significant either. This could also be because of the measure's limitations, although one might argue that teacher ratings should be more closely associated with educational attainment than other ratings of personality. The observed effect of childhood Dependability on Education was small regardless of whether IQ was residualised from teachers' individual characteristic ratings before creating the Dependability measure. Another possibility is that this effect was attenuated by the other childhood factors in the model. For example, Social Class showed a weak but significant correlation with Dependability, and a correlation of .29 with Education. This suggested that childhood Social Class acted as a cultural marker for an environment that supported educational attainment, as well as the establishment of lifelong dependable and healthy lifestyle habits in childhood, which contributed to health and wellbeing in older age. As Shanahan et al. (2014) and Damian et al. (2015) suggested, this might mean that the association between childhood personality and later‐life outcomes could be very different for individuals of higher or lower social class. However, we tested whether the effects of childhood Dependability were attenuated by Social Class or other variables in childhood by removing them from the models, and the coefficients for all paths from Dependability were still small and non‐significant.

We also observed that the effects of personality traits were generally in the expected direction, but were weaker than hypothesised, and weaker than previous reports of contemporaneous associations between personality traits and health in adulthood and older age (Gilhooly et al., 2007; Jokela et al., 2013; Gleason et al., 2014). However, these modest correlations with Health may again indicate that the effects of personality traits were partly obscured by other predictors, that is that the effects of early‐life predictors via Education were more important. This inference does tie with previous findings, such as those of Hampson et al. (2015), indicating that childhood conscientiousness, through educational attainment in young adulthood, contributed to establishing healthy habits that can be maintained throughout life and into older age without contributions from current conscientiousness. This also complements previous work suggesting that cumulative influences of early‐life factors on later‐life health outcomes are more important than other contemporaneous influences (Shanahan et al., 2014; Damian et al., 2015). When we included Wellbeing as an intermediary in the model of older‐age Health, the influences of early‐life variables were reduced, suggesting that Wellbeing partially mediated the full effects of early‐life factors on Health. With the reduced effects of early‐life variables, the contribution of Agreeableness to Health was also much greater. This effect must have been muted by the greater effects of early‐life variables in the separate model, which provided support for the idea that the mediatory effects of older‐age personality traits were partly obscured by early‐life predictors. Interestingly, the effect of Agreeableness on Health that became apparent when Wellbeing was included in the model was negative. This may seem counterintuitive, but is consistent with previous findings, for example that lower agreeableness was associated with better outcomes among older emergency room patients (Chapman et al., 2009).

Older‐age personality traits did, however, play important roles in both models of older‐age Wellbeing. This was expected, considering previous evidence of close associations between personality and wellbeing (Schmutte & Ryff, 1997; DeNeve & Cooper, 1998; Schimmack et al., 2002; Steel et al., 2008; Gale et al., 2013). Emotional Stability showed a particularly strong correlation with Wellbeing, and also (along with Extraversion in the second model of Wellbeing) mediated the effects of childhood IQ, Social Class and Education. This also supported previous work suggesting that emotional stability (or its opposite, neuroticism) is most closely associated with subjective wellbeing (Vittersø, 2001; Schimmack et al., 2002; Friedman et al., 2010; Brett et al., 2012). The involvement of older‐age personality traits was the main way in which models of Wellbeing differed from models of Health; early‐life variables predicted both outcomes similarly. Wellbeing was therefore closely related to personality in older age, as well as to Health and its lifelong predictors. Wellbeing could perhaps be seen as a result of personality and health—dependent on objective health outcomes, but also on how different personalities respond and contribute to such outcomes. Indeed, our model of Wellbeing via Health had more associations that were strong enough to achieve statistical significance and the model of Health via Wellbeing had the poorer fit. The poor fit of this model suggested that either unmeasured variables played important, possibly moderating, roles, or that the direction of effect between health and wellbeing was primarily the other way around. However, as above, our results, along with those of others assessing the relations between personality and wellbeing may simply reflect the overlap between the constructs of personality and wellbeing. We cannot infer that aspects of personality are involved in determining wellbeing, as these aspects of personality may also fall within the very definition of wellbeing. Importantly, we assessed subjective wellbeing, but used measures of both its cognitive and affective components.

### Other early‐life predictors

Although childhood Dependability played at best only minor roles in predicting older‐age Health and Wellbeing, childhood Social Class and particularly IQ were significant predictors of both outcomes, included in all four final models. This supported previous evidence that health and wellbeing outcomes depend on earlier cognitive ability (O'Toole & Stankov, 1992; Deary et al., 2004; Voracek, 2009; Calvin et al., 2011; Hagger‐Johnson et al., 2012; Wrulich et al., 2014) and social class (Montgomery & Carter‐Pokras, 1993; Fein, 1995; Lachman & Weaver, 1998; Stansfield et al., 1998; Hagger‐Johnson et al., 2012). Importantly, however, neither of these variables influenced Health or Wellbeing via its older‐age counterpart; instead, both variables exerted influence over later‐life outcomes via Education, which we had expected to act as an important mediator of all childhood predictors of older‐age Health and Wellbeing. This may be explained by education generally being associated with occupation of health‐related environments and participation in health‐related behaviours (Hampson et al., 2007; Shanahan et al., 2014; Damian et al., 2015). And if lifelong health behaviours do account for the association between Education and older‐age Health in this study, it would explain why the effects of other older‐age variables as mediators of the effects of early‐life variables or as additional predictors of older‐age Health were relatively small.

Finally, we consider the role of childhood Illness. This variable showed a direct negative association with older‐age Health, as might be expected, but did not influence Education or show any additional effects on other intermediary variables. However, removing childhood Illness from models of older‐age Health increased the weights of paths from childhood IQ and Social Class to Education, and from Education to older‐age Health. This suggests that childhood Illness was partially masking these effects, perhaps because those who were ill as children tended to be of lower IQ and Social Class. It may be that health was already dependent on these factors even in childhood, or that the IQ and Social Class of participants in childhood reflected that of their parents, whose education and established health behaviours influenced the health of the children they cared for. This would be an intergenerational transmission mechanism underlying older‐age health and wellbeing comparable to that proposed by Shanahan et al. (2014), focusing on the role of parental conscientiousness in the development of health behaviours.

## Conclusion

In conclusion, our results suggested that childhood dependability played relatively small roles in influencing older‐age health and subjective wellbeing, whereas other early‐life factors such as intelligence and social class were more important predictors. Older‐age Big Five personality traits were not strongly associated with older‐age health either, but showed strong associations with subjective wellbeing, although this may simply have been attributable to overlap between the assessments of personality and subjective wellbeing. The effects of childhood intelligence and social class on older‐age health and wellbeing were not simply through association with intelligence and social class in later life. Instead, these variables appeared to influence education, which had independent associations with health and subjective wellbeing decades later, most likely by promoting early establishment of habits involving health behaviours that have lifelong cumulative effects.

Our failure to find evidence of substantive influences of childhood personality in no way rules them out. Our dependability measure of childhood personality was narrow in scope and assessed by few items, rated by observers whose contact with those they were rating was largely limited to settings eliciting display of the rated characteristics through required performance of specific tasks, and who were also charged with rating the quality of those task performances. Its effects may also have been overshadowed by associations with childhood intelligence and social class. Furthermore, childhood dependability was only observed in the smaller of our two samples. Thus our results relating to this measure must at best be considered preliminary, and significant influences of childhood personality on older‐age health and wellbeing may well be observed in future studies using larger samples and better measures.

The differences we observed among the four different ways of modelling our data, as well as the differences we observed when certain variables were removed from models, also suggested that, to disentangle the complex relations among early‐life personality, intelligence, social class, education and other factors, and their individual effects on later‐life health and wellbeing, future studies will need to use both more precise measures—in particular, a much more comprehensive and established measure of childhood personality—and more frequent assessments. They will also need to be designed more specifically to test some of the possible explanations offered here and to test them against others offered in the literature. Our findings show that targeting early‐life factors such as personality, intelligence, social class and education may eventually lead to better health and wellbeing in later life, and studies following on from this one may tell us how best to do this.
